# Supplementary material for: COVID-19 Vaccine Confidence Mediates the Relationship between Health Literacy and Vaccination in a Diverse Sample of Urban Adults
Source: Vaccines (Basel). 2023 Dec 13;11(12):1848. doi: 10.3390/vaccines11121848 (PMC10747333; doi:10.3390/vaccines11121848)
Supplement: Supplementary file 1 [file vaccines-11-01848-s001.zip › vaccines-2738339-supplementary.pdf]

Supplementary Materials

Table S1.

Table S1: Mediation Model Estimates with Missingness Including Direct and Indirect Effects			
	Path	Estimate	95% Bootstrapped Confidence Intervals
Missingness	**Mediator ==> Vaccination**		
(Yes = 1; No = 0)	Missingness ==> Vaccination	-0.07	-0.75, 0.88
Health literacy	**Direct effects**		
(continuous)	Health literacy ==> Vaccination	0.00	-0.04, 0.04
Vaccine confidence (aVCI, continuous)	**Mediator ==> Vaccination**		
	aVCI ==> Vaccination	0.87*	0.42, 1.58
	**Mediator ==> Health literacy**		
	aVCI ==> Health literacy	0.04*	0.03, 0.06
	**Indirect effects on Vaccination**		
	Health literacy ==> aVCI ==> Vaccination	0.04*	0.02, 0.08

Table S1. Note. aVCI = Adapted Vaccine Confidence Index; Estimates are unstandardized. Starred (*) estimates indicate 95% confidence intervals not containing zero; Vaccine confidence is the mediator.

Appendix A
